# Lipoic Acid-Modified Oligoethyleneimine-Mediated miR-34a Delivery to Achieve the Anti-Tumor Efficacy

**DOI:** 10.3390/molecules26164827

**Published:** 2021-08-10

**Authors:** Yu Huang, Longxiang Wang, Yingxuan Chen, Haobo Han, Quanshun Li

**Affiliations:** Key Laboratory for Molecular Enzymology and Engineering of Ministry of Education, School of Life Sciences, Jilin University, Changchun 130012, China; huangyu1315@mails.jlu.edu.cn (Y.H.); wanglx1318@mails.jlu.edu.cn (L.W.); chenyu1317@mails.jlu.edu.cn (Y.C.)

**Keywords:** lipoic acid, oligoethyleneimine, miR-34a, anti-tumor efficacy, gene therapy

## Abstract

MiR-34a, an important tumor suppressor, has been demonstrated to possess great potential in tumor gene therapy. To achieve the upregulation of miR-34a expression level, an oligoethyleneimine (OEI) derivative was constructed and employed as the carrier through the modification with lipoic acid (LA), namely LA-OEI. In contrast to OEI, the derivative LA-OEI exhibited superior transfection efficiency measured by confocal laser scanning microscopy and flow cytometry, owing to rapid cargo release in the disulfide bond-based reduction sensitive pattern. The anti-proliferation and anti-migration effects were tested after the miR-34a transfection to evaluate the anti-tumor response, using human cervical carcinoma cell line HeLa as a model. The delivery of LA-OEI/miR-34a nanoparticles could achieve obvious anti-proliferative effect caused by the induction of cell apoptosis and cell cycle arrest at G1 phase. In addition, it could inhibit the migration of tumor cells via the downregulation of MMP-9 and Notch-1 level. Overall, the LA-OEI-mediated miR-34a delivery was potential to be used as an effective way in the tumor gene therapy.

## 1. Introduction

Cancer has been accepted as a leading cause of death in humans [1], and conventional therapeutic strategies based on radiotherapy, chemotherapy and surgery still remain a formidable challenge for the recurrence of cancer [2,3]. Except for these routes, gene therapy has been widely investigated in which exogenous nucleic acids were used as therapeutics to suppress the oncogenes and trigger the anti-proliferation and anti-migration effects of tumors [4,5]. Thus, it has been identified to be an important approach for the cancer treatment. MicroRNAs (miRNAs) as non-coding oligonucleotides, could post-transcriptionally manipulate the gene expression by targeting the 3′-UTR of intracellular mRNAs [6,7,8]. Dysregulation of miRNAs has been elucidated to be related to the proliferation and metastasis of cancers, which has been recognized as a marker of cancer progression [6]. Particularly, miR-34a has been confirmed to be a well-defined tumor suppressor which could persuade the cell apoptosis, the cell cycle arrest at G1 phase and also restrain the migration of tumor cells by decreasing the expression of Bc1-2, Notch-1, survivin, CD44 and the cyclin family [9,10,11,12,13].

Since miRNA is susceptible to pH or enzymes in human bodies when delivered in vivo, an effective and safe carrier is highly required to realize the miR-34a transfection in tumor tissues [14,15]. Viral-based gene carriers including adenovirus, adeno-associated virus and retrovirus have shown inherent shortcomings such as unsatisfied immunogenicity and risk of carcinogenesis [16,17]. Unlike viral vectors, cationic polymers have been demonstrated to possess a series of advantages including limited immunogenicity, high loading capacity and ease of production [18,19,20,21]. Among cationic polymers, polyethyleneimine (PEI) with molecular weight of 25,000 g/mol possessed high transfection efficiency, and thus it has been accepted as a gold standard in polymeric gene carriers [22,23,24]. However, the relatively high toxicity of PEI25K limited its clinical application in tumor gene therapy. In comparison to PEI25K, oligoethyleneimine (OEI) exhibited favorable biocompatibility but its transfection efficiency was relatively lower [25,26,27]. Thus, extensive efforts have been devoted to improve its transfection efficiency through the modification with other molecules such as β-cyclodextrin [26], acetal [28], alkyl acrylates [29], poly(β-amino ester) [30] and polypropylenimine dendrimers [31]. Previously, we developed a biodegradable OEI1800 derivative through the modification of lipoic acid (LA) with a disulfide bond, and the derivative LA-OEI showed favorable transfection efficiency due to its reduction-sensitive capability to accelerate the rapid intracellular release of therapeutic genes, as the disulfide bond of LA could be easily cleaved by the reduction environment in the cytosol [32].

In the present study, LA-OEI was synthesized and used as a vector to facilitate the miR-34a transfection, in which the delivery efficiency was evaluated using flow cytometry (FACS) and confocal laser scanning microscopy (CLSM). Furthermore, the anti-tumor efficacy induced by LA-OEI/miR-34a transfection was investigated using human cervical carcinoma cell line HeLa as a model.

## 2. Results and Discussion

### 2.1. Synthesis and Characterization of LA-OEI

**Figure 1:**
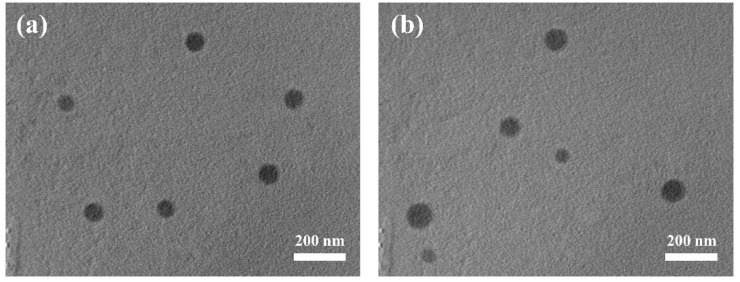
The morphology analysis of nanoparticles using TEM. (**a**) LA-OEI/miR-34a; (**b**) OEI/miR-34a. The scale bar is 200 nm.

The derivative LA-OEI was prepared through the LA’s modification on OEI1800 according to our previous study [32,33], and the product structure has been proven by ^1^H NMR. Then the LA-OEI/miR-34a nanoparticles were constructed with different mass ratios, and the gel retardation assay was carried out to assay the carrier’s binding ability with miR-34a (Figure S1 in Supplementary Materials). Apparently, the carriers OEI and LA-OEI both exhibited favorable ability to bind and condense miR-34a into nanoparticles, as they could achieve the complete retardation of miR-34a at mass ratios of 0.6 and 0.8, respectively. The subtle difference demonstrated the decreased binding ability of LA-OEI towards miR-34a owing to the modification of cationic amine groups with LA. Since miRNAs are susceptible to pH or enzymes in vivo, the protective effect of LA-OEI was conducted through the incubation of LA-OEI/miR-34a nanoparticles with RNase A and 50% fetal bovine serum (FBS), respectively, which were treated with heparin and analyzed through agarose gel electrophoresis (Figure S2). Free miR-34a could be easily degraded by the treatment with 50% FBS or RNase A, while clear bands of miR-34a were found for LA-OEI/miR-34a groups after the treatment with 50% FBS or RNase A, indicating that the derivative LA-OEI could protect miR-34a against the degradation of RNase A and 50% FBS. Thus, the protective effect of LA-OEI could efficiently stabilize miR-34a, which was potential to further prolong the half-life of miR-34a when it was used in vivo. Next, the morphology of LA-OEI/miR-34a nanoparticles was observed through transmission electron microscopy (TEM) (Figure 1). Similar spherical structure could be clearly observed for both LA-OEI/miR-34a and OEI/miR-34a nanoparticles, indicating that these two carriers could condense miR-34a into stable nanoparticles through electrostatic interactions with diameter values of <200 nm. Moreover, the hydrodynamic diameter and zeta potential of nanoparticles were measured at various carrier/miR-34a ratios (Table S1). The hydrodynamic diameter of OEI/miR-34a and LA-OEI/miR-34a nanoparticles showed a decreased tendency with the improvement of mass ratio, whereas the zeta potential values gradually increased. Meanwhile, in comparison to OEI/miR-34a nanoparticles, LA-OEI/miR-34a nanoparticles exhibited a relatively smaller size and higher positive charge density, which was beneficial for achieving the in vitro transfection. Particularly, LA-OEI/miR-34a nanoparticles at a mass ratio of 5.0 possessed hydrodynamic diameter and zeta potential values of 123.4 ± 2.1 nm and +47.0 ± 0.7 mV, respectively. The favorable diameter (<200 nm) was beneficial to achieve the cellular uptake of nanoparticles in an endocytosis manner, and also potential to be used as a promising gene delivery system in vivo. Moreover, the positively charged state could further facilitate the interaction with cell membrane of negative zeta potential, thereby obtaining ideal cellular uptake of nanoparticles.

### 2.2. In Vitro Transfection Efficiency of LA-OEI/miR-34a Nanoparticles

**Figure 2:**
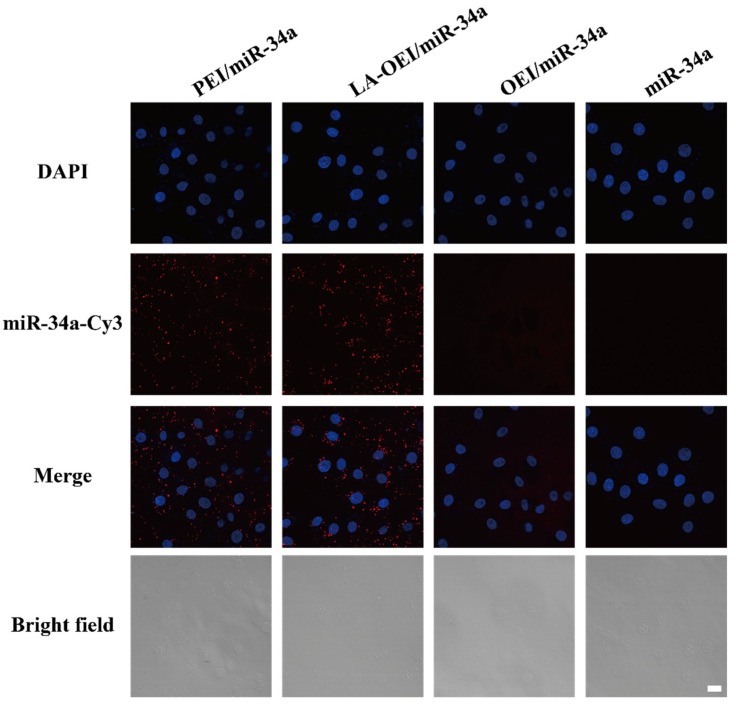
The transfection efficiency analysis of free miR-34a, PEI/miR-34a, LA-OEI/miR-34a and OEI/miR-34a nanoparticles in HeLa cells through CLSM images. The scale bar is 20 μm.

**Figure 3:**
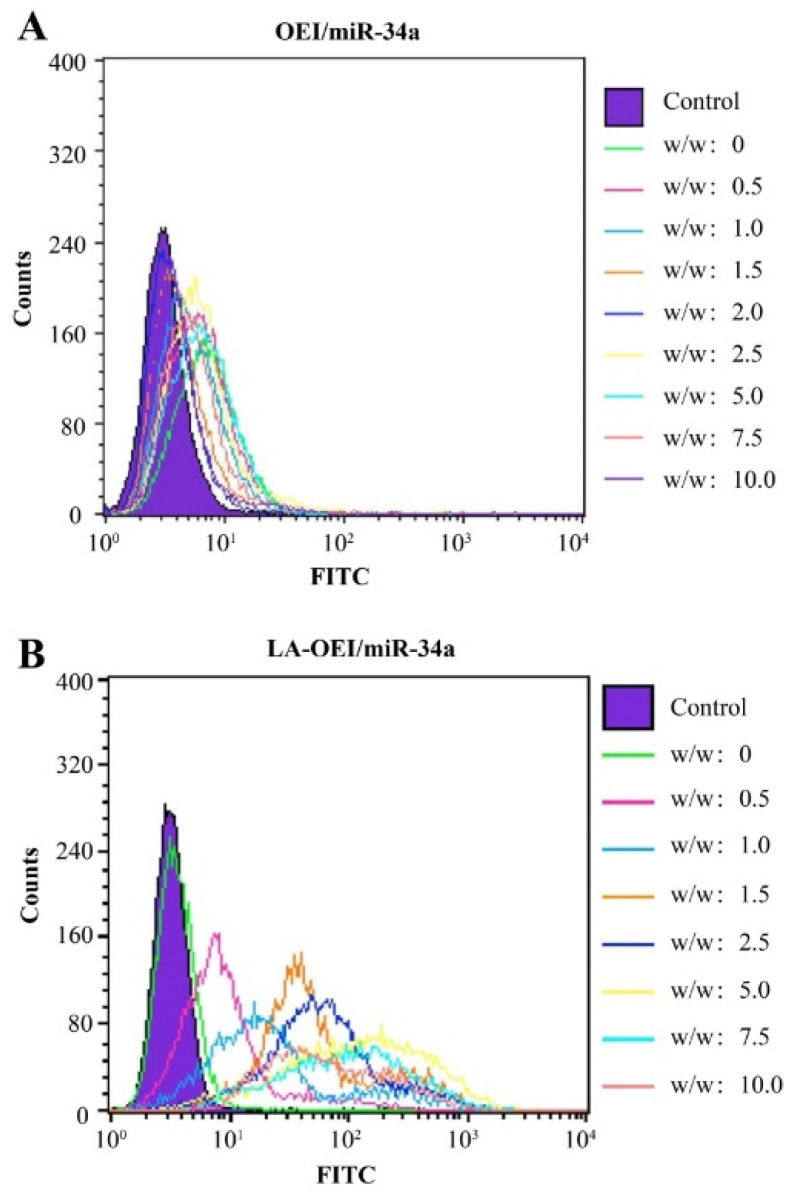
The transfection efficiency of nanoparticles at different mass ratios using FITC-labeled miR-34a. (**A**) OEI/miR-34a; (**B**) LA-OEI/miR-34a.

Subsequently, the in vitro transfection efficiency analysis of LA-OEI/miR-34a nanoparticles was performed through FACS and CLSM. There was no obvious red fluorescence observed in the transfection with Cy3-labeled miR-34a group, suggesting that HeLa cells could not achieve the cellular uptake of free miR-34a (Figure 2). Moreover, limited fluorescence signal has been obtained after the OEI/miR-34a transfection, attributing to the low transfection efficiency of OEI. Remarkably, LA-OEI/miR-34a group showed obvious red fluorescence demonstrating the LA-OEI’s superior transfection efficiency. Meanwhile, the transfection ability could be achieved at a similar level to PEI/miR-34a nanoparticles. Furthermore, the transfection efficiency of different nanoparticles at different mass ratios was determined through FACS (Figure 3 and Figure S3). Consistently, limited transfection efficiency of OEI/miR-34a nanoparticles could be detected, while LA-OEI-mediated miR-34a delivery achieved higher transfection efficiency at an identical mass ratio. In particular, LA-OEI/miR-34a nanoparticles showed remarkable transfection ability when the mass ratio reached 5.0, with over 90% transfection efficiency. In addition, in comparison to Lipofectamine 2000, LA-OEI showed a relatively higher transfection efficiency using Cy5-labeled miR-34a (Figure S4). These results were mainly caused by the intermolecular cross-linking mediated by the disulfide bonds of LA, leading to an enhanced positive charge density of LA-OEI. Moreover, the reduction-sensitive ability of LA-OEI provided a rapid intracellular gene release owing to the cleavage of disulfide bonds by the high reduction environment in the cytosol, resulting in excellent transfection ability. Previous reports have shown that the modification with LA could enhance the lipophilicity of OEI, thereby improving the cellular uptake of LA-OEI/miR-34a nanoparticles [34]. However, there were no obvious differences in the cellular uptake observed for the carriers OEI and LA-OEI (Figure S5). Thus, the improved transfection efficiency of LA-OEI/miR-34a nanoparticles was not attributed to the change of cellular uptake ability, but highly associated with the enhanced miR-34a release owing to the reduction-sensitive characteristic of LA-OEI. The OEI’s limited transfection efficiency was probably caused by the trap of OEI/miR-34a nanoparticles in the endosomes after their successful endocytosis.

### 2.3. Anti-Proliferative Effect of LA-OEI/miR-34a Nanoparticles

**Figure 4:**
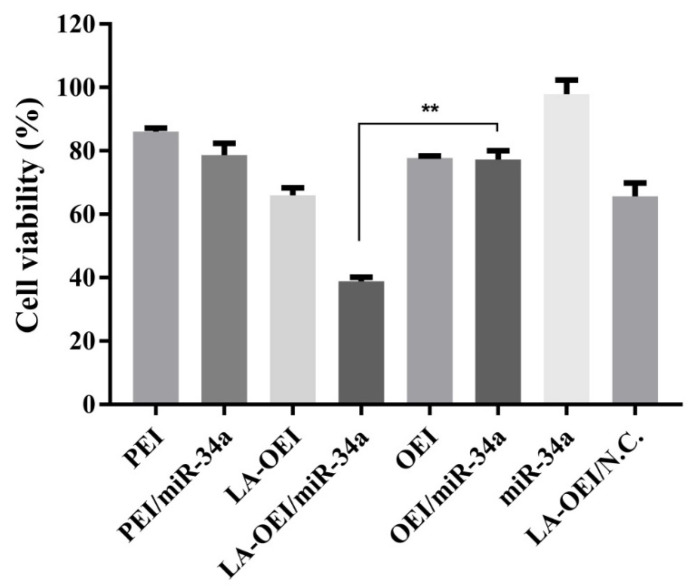
The anti-proliferative effect of HeLa cells after the treatment with different nanoparticles. Data were presented as the mean value ± SD of triplicate experiments (** *p* < 0.01).

Furthermore, we explored the anti-proliferation effect of LA-OEI/miR-34a nanoparticles using 3-(4,5-dimethylthiazol-2-yl)-2,5-diphenyltetrazolium bromide (MTT) assay. As elucidated in Figure 4, LA-OEI exhibited a relatively higher cytotoxicity than OEI which was probably attributed to the improved molecular weight owing to the disulfide bond-mediated intermolecular cross-linking of OEI. Remarkably, LA-OEI/miR-34a nanoparticles could achieve obvious anti-proliferative effect in HeLa cells with an inhibitory ratio of 32.4%, much higher than PEI/miR-34a nanoparticles (8.2%). Nevertheless, no obvious anti-proliferative effect was observed in the OEI-mediated miR-34a transfection due to its poor transfection efficiency. Furthermore, the anti-proliferative effect of LA-OEI/miR-34a nanoparticles was evaluated using live/dead staining assay (Figure S6), in which the viable and dead cells emitted green (owing to calcein AM) and red fluorescence (owing to ethidium homodimer), respectively. Apparently, a large population of dead cells were produced in the LA-OEI/miR-34a group, whereas OEI/miR-34a nanoparticles did not induce the cell death efficiently. In addition, the anti-proliferative effect after the miR-34a transfection was assayed using the formation ability of the cell colony (Figure S7). The LA-OEI/miR-34a nanoparticles exhibited significant inhibition of colony formation in HeLa cells, stronger than in the PEI/miR-34a group. In a word, LA-OEI could efficiently facilitate the intracellular delivery of miR-34a and further trigger the anti-proliferative effect in HeLa cells.

**Figure 5:**
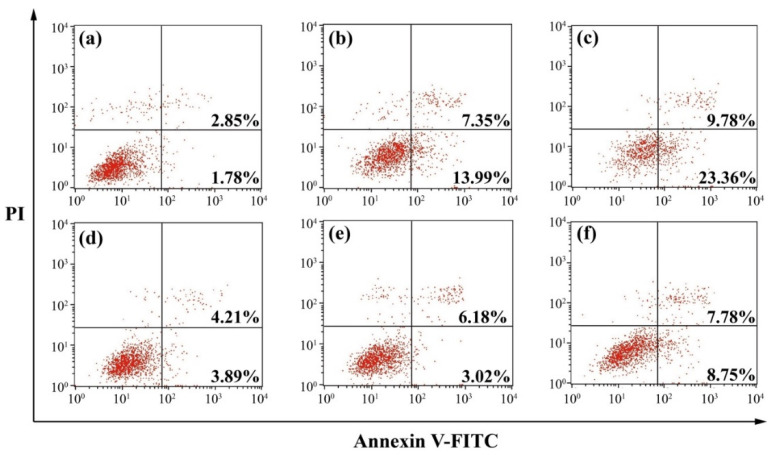
Cell apoptosis analysis after the miR-34a transfection. The transfected cells were stained with Annexin V-FITC and PI and analyzed by FACS: (**a**) control, (**b**) PEI/miR-34a, (**c**) LA-OEI/miR-34a, (**d**) OEI/miR-34a, (**e**) miR-34a, and (**f**) LA-OEI/N.C.

**Figure 6:**
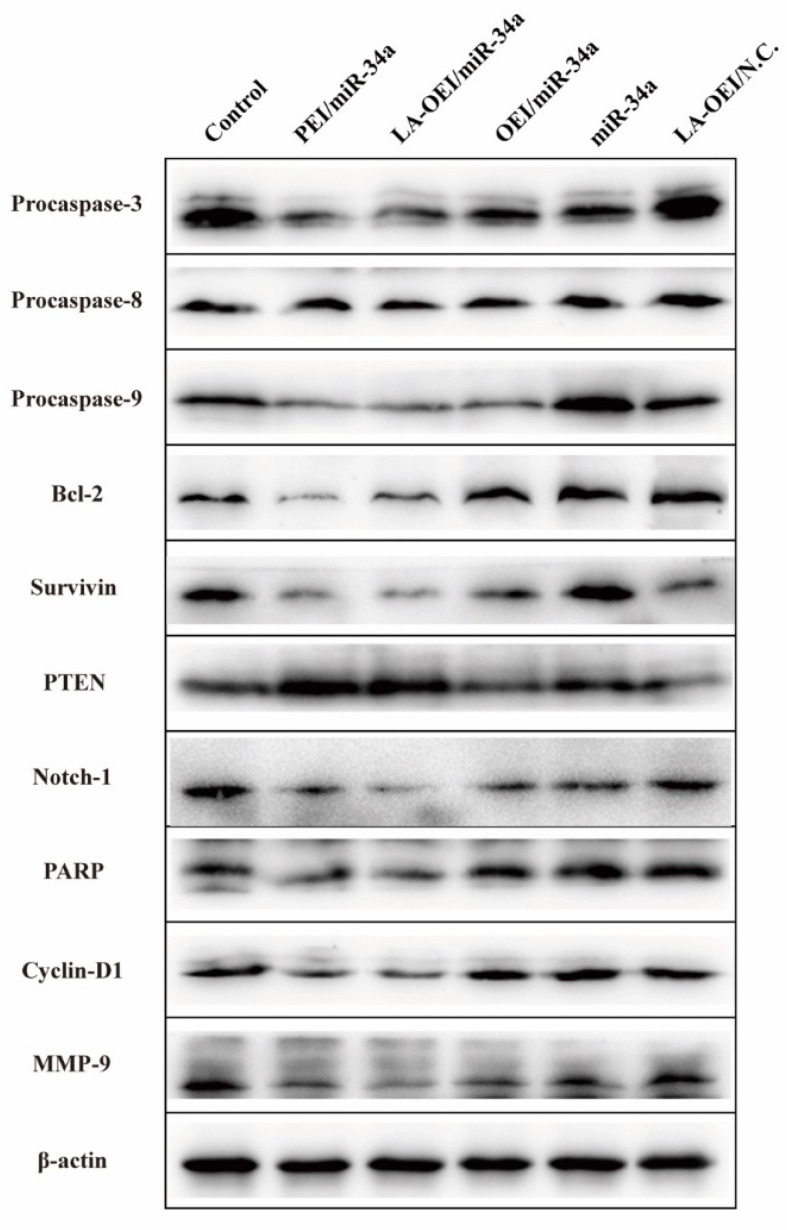
The protein expression level analysis after the miR-34a transfection. The HeLa cells were treated with different nanoparticles and the related proteins were analyzed using Western blotting.

To understand the mechanism for the anti-proliferative effect triggered by LA-OEI/miR-34a nanoparticles, the cell apoptosis was analyzed through the Annexin V-FITC/PI dual staining of the transfected cells using FACS (Figure 5). Clearly, no significant cell apoptosis was detected in free miR-34a and OEI/miR-34a groups with early apoptotic ratios of 3.02% and 3.89%, respectively. In contrast, miR-34a transfection using LA-OEI and PEI as carriers could realize obvious early apoptosis. Meanwhile, LA-OEI/miR-34a nanoparticles could achieve much stronger cell apoptosis than PEI/miR-34a nanoparticles (23.36% vs. 13.99%), which was attributed to the enhanced intracellular delivery of miR-34a. However, the reactive oxygen species (ROS) level showed that the transfection of nanoparticles did not influence the intracellular ROS level, using DCFH-DA as a probe (Figure S8). Thus, the induction of cell apoptosis by miR-34a transfection was not associated with the improvement of intracellular ROS level, and the apoptosis-inducing mechanism needed to be systematically discussed. Subsequently, the expression level of proteins related to the cell apoptosis was detected through Western blotting (Figure 6). The procaspase-3 level was dramatically decreased after the delivery of miR-34a using PEI and LA-OEI as carriers, indicating the precursor cleavage of procaspase-3 and the activation of caspase-3. After the caspase-3 activation, PARP (the specific substrate of caspase-3) was cleaved, which was essential for the occurrence of cell apoptosis. In comparison to the control group, down-regulated expression of PARP could be observed in LA-OEI- and PEI-mediated miR-34a delivery. Additionally, compared with procaspase-8, there was a significant down-regulation of procaspase-9 obtained after the delivery of miR-34a using LA-OEI and PEI as carriers, suggesting the cleavage of procaspase-9 to activate the caspase-9. Consistently, the relative activities of caspae-3 and -9 were measured to be increased after the treatment with these two nanoparticles (Figure S9). Meanwhile, no obvious activation of caspase-8 has been found for the cells treated with LA-OEI/miR-34a and PEI/miR-34a nanoparticles. The activation of caspase-9 indicated that the carriers-mediated miR-34a delivery induced the cell apoptosis through the mitochondria-dependent signaling route. Interestingly, the miR-34a delivery could reduce the expression level of survivin and Bcl-2. As known, Bcl-2 and survivin have been identified to be over-expressed in a large number of cancer cells and play a critical role in the tumorigenesis and therapeutic resistance in the cancer treatment [35,36]. Thus, the decreased expression of Bcl-2 and survivin induced by miR-34a transfection could not only enhance the apoptotic effect but also hinder the drug-resistance of cancer cells. Moreover, the miR-34a transfection could improve the PTEN expression level. Since PTEN has been proposed as a well-characterized tumor suppressor that was mutated or deficient in many human cancers [37], the miR-34a-mediated restoration of PTEN could inhibit the cell proliferation and thereby enhance the therapeutic efficacy.

**Figure 7:**
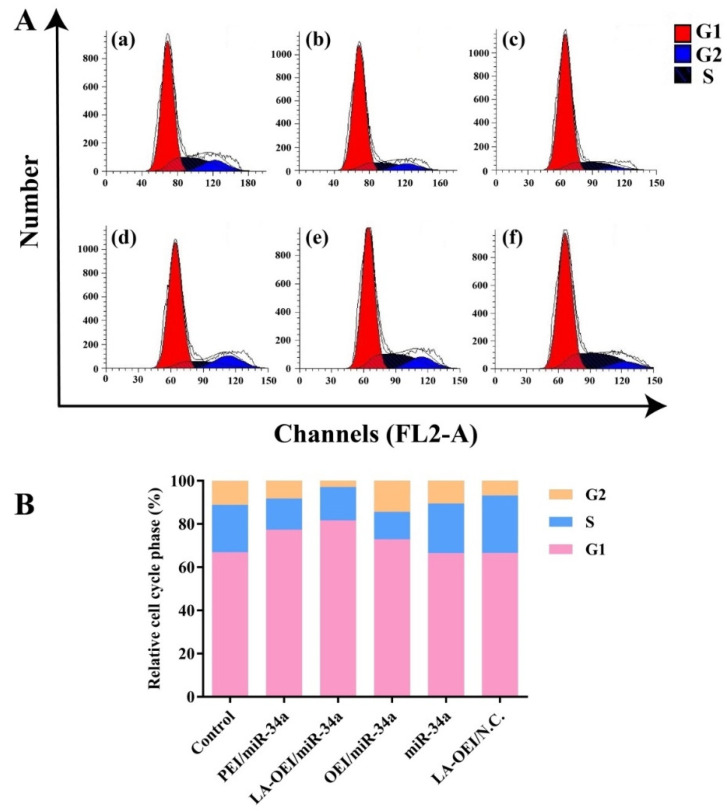
The cell cycle arrest analysis of HeLa cells after miR-34a transfection. (**A**) the cell cycle analysis of HeLa cells induced by different nanoparticles using FACS: (**a**) control, (**b**) PEI/miR-34a, (**c**) LA-OEI/miR-34a, (**d**) OEI/miR-34a, (**e**) miR-34a and (**f**) LA-OEI/N.C. (**B**) Quantitative analysis of the cell cycle.

Except for the cell apoptosis, the arrest of cell cycle was another important reason leading to the inhibition of cell proliferation. Compared to the control group, a large population of cells could be arrested at G1 phase after the miR-34a transfection, suggesting the effective arrest of cell cycle at G1 phase (Figure 7 and Figure S10). In particular, the G1 phase ratio of 82.50% was obtained for the LA-OEI/miR-34a nanoparticles, which was much higher than the miR-34a transfection by PEI (78.30%) and OEI (74.58%). Meanwhile, there was no significant improvement of G1 phase ratio in free miR-34a and LA-OEI/N.C. This phenomenon probably relied on the reduced Cyclin D1 level after the miR-34a transfection (Figure 6), as Cyclin D1 was accepted to participate in the G1/S transition [38]. In summary, LA-OEI/miR-34a transfection could achieve a stronger anti-tumor response than OEI- and PEI-mediated miR-34a delivery, in a manner of triggering the cell apoptotic effect and the cell cycle arrest at G1 phase. Thus, the intracellular delivery of miR-34a using LA-OEI as a carrier could potentially be an important route to achieve the cancer gene therapy.

### 2.4. Anti-Migration Effect of LA-OEI/miR-34a Nanoparticles

**Figure 8:**
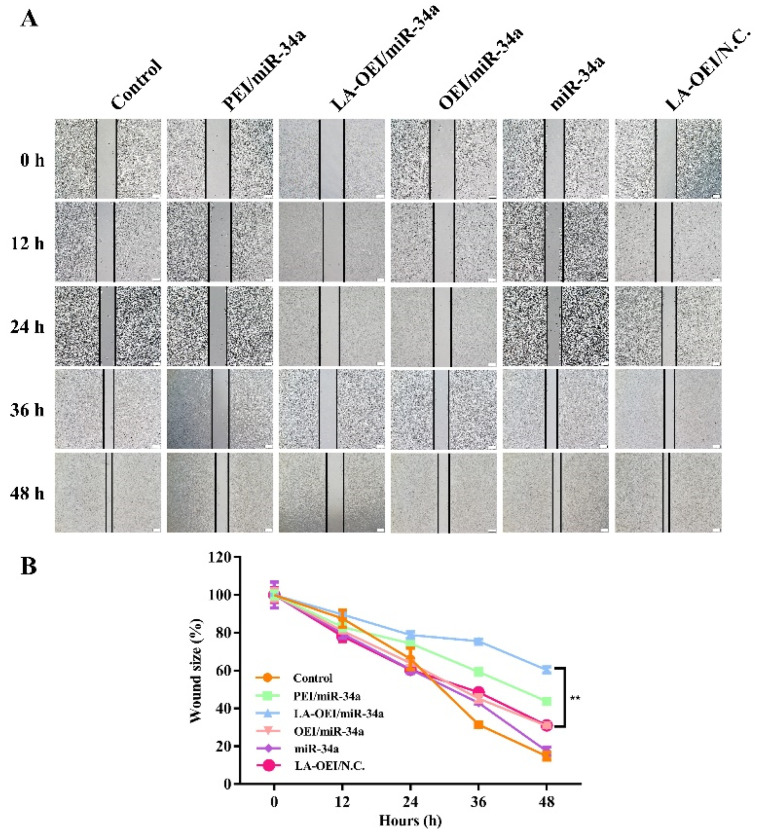
The anti-migration effect of HeLa cells after the miR-34a using wound healing assay. (**A**) the scratch observation of HeLa cells after the treatment with different nanoparticles. The scale bar is 200 μm. (**B**) Quantitative analysis of wound size, in which data were presented as the mean value ± SD of triplicate experiments (** *p* < 0.01).

**Figure 9:**
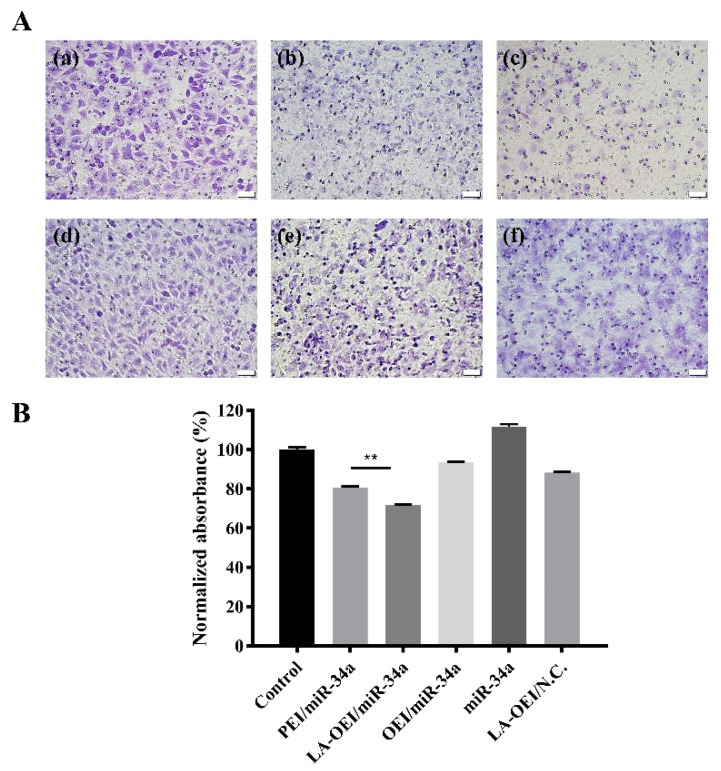
The anti-migration effect of HeLa cells after the miR-34a transfection using Transwell migration assay. (**A**) The staining with 0.1% crystal violet for the cells which have migrated to the lower surface of membrane: (**a**) control, (**b**) PEI/miR-34a, (**c**) LA-OEI/miR-34a, (**d**) OEI/miR-34a, (**e**) miR-34a and (**f**) LA-OEI/N.C. The scale bar is 50 μm. (**B**) Quantitative analysis of the cells which have migrated to the lower surface of membrane. Data were presented as the mean value ± SD of triplicate experiments (** *p* < 0.01).

The migration ability of cancer cells was critical for the metastasis and further recurrence of malignancy. Thus, we detected the anti-migration effect after the miR-34a transfection using wound healing and Transwell migration assays. As shown in Figure 8, the wound size was measured after the transfection of different nanoparticles within 48 h. In this regard, we found that the scratch was rapidly healed in the control and free miR-34a groups within 48 h. In contrast, larger wound size could be retained when the cells were transfected with PEI/miR-34a and LA-OEI/miR-34a nanoparticles which suggested that these two nanoparticles could obviously trigger the anti-migration effect of cancer cells. Further, Transwell migration assay was employed to monitor the anti-migration and anti-invasion effect induced by different nanoparticles harboring miR-34a (Figure 9). The number of cells which have migrated to the lower chamber was significantly reduced in PEI- and LA-OEI-mediated miR-34a transfection groups, where LA-OEI/miR-34a nanoparticles were more effective to inhibit the migration and invasion of cells. Basically, the matrix metalloproteinase (MMP) family, particularly MMP-9, was identified to executive a key function in the metastasis by degrading the extracellular matrix in the process of tissue remodeling [39]. The MMP-9 expression decreased after the miR-34a transfection (Figure 6), which was probably an important reason for the suppression of cell migration. Moreover, the anti-migration effect of miR-34a delivery was considered to be associated with the down-regulated expression of Notch-1, another key factor involving in the metastasis procedure [40]. Taken together, these results meant that the miR-34a transfection could efficiently suppress the migration and invasion of cancer cells by blocking MMP-9 and Notch-1 signaling pathways, potentially reducing the recurrence of malignancies.

## 3. Materials and Methods

### 3.1. Materials

The miR-34a (sense: 5′-UGGCAGUGUCUUAGCUGGUUGU-3; antisense: 3′-AACCAGCUAAGACACUGCCAUU-5′) and its Cy3- and FITC-labeled forms were provided by GenePharma (Suzhou, China). Phosphate-buffered saline (PBS) and Dulbecco’s modified Eagle’s medium (DMEM) were obtained from Gibco (Grand Island, NE, USA). FBS was purchased from Kangyuan Co. (Beijing, China). MTT, RNase A, 4,6-diamidino-2-phenylindile (DAPI) and heparin were acquired from Amersco (Solon, OH, USA). The caspase-3, -8 and -9 activity assay kits were provided by Promega (Madison, WI, USA). The live/dead cell staining kit was obtained from ThermoFisher (Eugene, OR, USA). The apoptosis and cell cycle detection kits were purchased from BestBio (Shanghai, China). The primary antibodies for Bcl-2, survivin, PTEN, Notch-1, PARP, Cyclin-D1, MMP-9, procaspase families and β-actin, and the horseradish peroxidase (HRP)-conjugated secondary antibody were obtained from Abcam (Shanghai, China).

### 3.2. Preparation and Characterization of LA-OEI

According to our previous report [32,33], the derivative LA-OEI was prepared by the LA’s modification on OEI1800. Briefly, 27.56 mg dicyclohexylcarbodiimide (DCC) and 7.69 mg N-hydroxysuccinimide (NHS) were added into 5 mL dichloromethane harboring 4.6 mg LA in a round-bottom flask under nitrogen. After stirring for 24 h, the sample was filtrated to discard the precipitate, and the solution was added dropwise to 30 mL dichloromethane containing 100 mg OEI1800 and stirred for an additional 24 h. After the treatment in cooling diethyl ether, the precipitate was collected through the centrifugation (8000 rpm, 15 min) and then lyophilized to obtain the product LA-OEI. The hydrodynamic diameter and zeta potential of nanoparticles were measured by Malvern Nano ZS90 Zetasizer (Malvern, UK). The nanoparticles’ morphology was observed by HITACHI-H800 TEM at an accelerating voltage of 200 kV.

### 3.3. Gel Retardation Assay

The nanoparticles were prepared by incubating the carriers (OEI and LA-OEI) and miR-34a at a series of mass ratios (0.1–1.0) for 30 min and then analyzed using 2% agarose gel electrophoresis in Tris-acetate-EDTA buffer (80 V, 15 min). In addition, the nanoparticles were treated with RNase A solution (10 μg/mL) and 50% FBS at 37 °C for 3 h, respectively. Then the protective effect against the degradation of miR-34a was detected using 2% agarose gel electrophoresis as described above.

### 3.4. In Vitro miR-34a Transfection

The transfection efficiency of LA-OEI/miR-34a nanoparticles was measured by FACS analysis. Briefly, HeLa cells were cultured overnight at an initial density of 1.5 × 10^5^ cells/well in 6-well plates harboring 2 mL of 10% FBS-containing DMEM in each well. The collected cells were washed with PBS twice and treated with LA-OEI/FITC-miR-34a nanoparticles (mass ratios of 1.0–10.0) in FBS-free medium for 6 h. Then the cells were detected on a FACS Calibur (BD Biosciences, San Jose, CA, USA). Further, the endocytosis of LA-OEI/miR-34a nanoparticles was observed by CLSM, in which HeLa cells were cultured on a sterilized glass slide in a 6-well plate overnight (initial density: 1.0 × 10^5^ cells/well). Through the treatment with nanoparticles as described above, the cells were rinsed with PBS twice and fixed in 4% paraformaldehyde for 15 min. After being stained with DAPI solution, the cells were analyzed on LSM710 instrument (Carl Zeiss Microscopy LLC, Jena, Germany).

### 3.5. Anti-Proliferative Effect of miR-34a Transfection

The anti-proliferative effect induced by LA-OEI/miR-34a nanoparticles was evaluated in HeLa cells by MTT assay. The cells were seeded in 96-well plates (initial density: 8.0 × 10^3^ cells/well). After the culture at 37 °C for 12 h, the cells were subjected to the treatment with LA-OEI/miR-34a nanoparticles in FBS-free medium for 6 h (mass ratio of 5.0, 2 μg/mL miR-34a). The cells were continued to grow in 10% FBS-containing DMEM for 48 h, and then 20 μL of MTT solution (5 mg/mL in PBS) was used to treat the cells for 4 h. The formed crystals were dissolved using 150 μL dimethyl sulfoxide, and the optical density at 492 nm was measured using GF-M3000 microplate reader (Weifang, China). Finally, the cell viability was calculated to be the ratio of optical density values of the sample to the control group.

### 3.6. Cell Apoptosis Analysis

The cells were first inoculated in 6-well plates (density: 2.0 × 10^5^ cells/well) and cultured in 10% FBS-containing DMEM overnight, and then transfected with 2 μg/mL miR-34a in FBS-free medium for 6 h and in 10% FBS-containing DMEM for 48 h. The harvested cells were rinsed with PBS twice and subjected to the Annexin V-FITC and PI staining according to the apoptosis detection kit’s instructions. In addition, the cells after the treatment with nanoparticles for 48 h were stained with DCFH-DA probe (10 μM) for 30 min according to the ROS detection kit’s instructions from Beyotime Biotechnology Co. (Jiangsu, China). The apoptosis and ROS level analysis were conducted on a FACS Caliber (BD Bioscience, Mountain View, CA, USA).

### 3.7. Live/Dead Cell Staining

The cell culture and miR-34a transfection were conducted as described in the Section 3.6. The harvested cells were washed with PBS twice, treated with live/dead reagents at room temperature for 20 min in the dark according to the manufacturer’s instructions and observed on an IX71 fluorescence microscopy (Olympus, Tokyo, Japan).

### 3.8. Cell Colony Formation Assay

The cell culture and miR-34a transfection were conducted as described in the Section 3.6. The collected cells were re-seeded in 6-well plates at a density of 3.0 × 10^3^ cells/well and incubated at 37 °C for 4 days, and the cell colony was stained with 0.1% crystal violet and observed by IX71 fluorescence microscopy (Olympus, Tokyo, Japan). In addition, the dyed cells were dissolved in 33% acetic acid after washing with PBS twice, and the optical density at 578 nm was detected using GF-M3000 microplate reader (Weifang, China).

### 3.9. Cell Cycle Arrest Analysis

The cell culture and miR-34a transfection were carried out as described in Section 3.6. The collected cells were fixed at −20 °C in the dark for 1 h using 75% cold ethanol, and RNase solution was used to treat the cells at 37 °C for 30 min. Finally, the staining with PI solution was performed at 4 °C for 30 min based on the cell cycle detection kit’s instructions, and the cell cycle was measured on a FACS Caliber (BD Bioscience, Mountain View, CA, USA).

### 3.10. Western Blotting Assay

The cell culture and miR-34a transfection were conducted as described in the Section 3.6. The transfected cells were harvested, washed with cold PBS twice and disrupted with RIPA lysis buffer on ice for 2 h. The lysates were centrifuged at 12,000 rpm for 10 min to collect the supernatant, in which the protein concentration was measured by BCA detection kit. Next, an equal amount of protein was subjected to SDS-PAGE and transferred to PVDF membrane by electroblotting. The membrane was blocked with PBST (0.1% Tween-20 in PBS) containing 5% skimmed milk at room temperature for 1 h and then incubated with the desired antibodies at 4 °C overnight. After washing with PBST twice, the membrane was incubated with the HRP-labeled secondary antibody at room temperature for 1 h. Finally, the membrane was visualized by enhanced chemiluminescence and detected on a Tanon 2500 imaging system (Shanghai, China) after washing with PBST.

### 3.11. Caspase-3, -8 and -9 Activities Analysis

The cell culture and miR-34a transfection were conducted as described in the Section 3.6, and the activities of caspase-3, -8 and -9 were determined by the corresponding activity assay kits. The transfected cells were disrupted with the lysis buffer supplied in the kits and incubated with the individual substrates for 2 h. The relative activities of caspase-3, -8 and -9 were monitored by measuring the luminescence signal using a Synergy HTX multi-mode microplate reader (BioTek, Winooski, VT, USA).

### 3.12. Anti-Migration Effect of miR-34a Transfection

The LA-OEI/miR-34a nanoparticles’ anti-migration effect was evaluated by wound scratch and Transwell migration assays. In the wound scratch assay, the cells were inoculated in 6-well plates at a density of 5.0 × 10^5^ cells/well and cultured in 2 mL of 10% FBS-containing DMEM to obtain a confluence of 90%. Then mechanical scratch was generated using a sterile pipette tip in the cell monolayer, and the cells were treated using different nanoparticles in FBS-free DMEM (2 μg/mL miR-34a in each group). Subsequently, the cells were cultured in DMEM containing 10% FBS for different time and detected using an IX71 fluorescence microscopy (Olympus, Tokyo, Japan) to capture the wound area. In the Transwell migration assay, the cell culture and miR-34a transfection were performed as described in Section 3.6. The cells after the transfection (1.0 × 10^4^ cells) were added in the upper part of Transwell chamber with 8-μm pores (Costar, Corning, NY, USA) harboring 200 μL of FBS-free DMEM, while 600 μL of 10% FBS-containing DMEM was placed into the lower part of the chamber. After 24 h, the mechanical wiping was performed to remove the non-migrated cells on the upper part of membrane. The cells migrating to the lower membrane surface were fixed in 75% cold ethanol and stained with 0.1% crystal violet, and the anti-migration effect was evaluated by IX71 fluorescence microscopy (Olympus, Tokyo, Japan).

### 3.13. Statistical Analysis

All the data were described as mean value ± standard deviation (SD) and analyzed by one-way ANOVA complemented with Student’s *t*-test, using SPSS Statistics 23.0. The statistical significance of differences was considered to be * *p* < 0.05 and ** *p* < 0.01 (n.s., not significant).

## 4. Conclusions

In conclusion, an efficient OEI derivative was constructed through the modification with LA and further used as a vector to realize the miR-34a transfection in cancer cells. Enhanced transfection efficiency could be obtained in LA-OEI/miR-34a nanoparticles, which was likely to be induced by the improved density of positive charge owing to the intermolecular crosslinking and the reduction-responsive gene release mediated by the disulfide bond. Correspondingly, the LA-OEI/miR-34a transfection could obviously induce the anti-proliferative effect, which was caused by the simultaneous activation of cell apoptosis and cell cycle arrest at G1 phase. Moreover, LA-OEI/miR-34a nanoparticles showed a favorable ability to suppress the cell migration and invasion by down-regulating the expression level of MMP-9 and Notch-1. In a word, the LA-OEI-mediated miR-34a transfection has been identified to possess excellent anti-tumor efficacy, potentially providing a promising platform to achieve the gene transfection in cancer treatment.

## Supplementary Materials

The following are available online, Figure S1: The miR-34a binding and condensation ability of different carriers using the gel retardation assay. A: OEI/miR-34a nanoparticles; B: LA-OEI/miR-34a nanoparticles, Figure S2: The protective effect of LA-OEI from the degradation of miR-34a. A: 50% FBS; B: RNase A, Figure S3: Quantitative analysis for the transfection efficiency of nanoparticles at different mass ratios based on FITC positive cells. A: OEI/miR-34a; B: LA-OEI/miR-34a. Data were presented as the mean value ± SD of triplicate experiments, Figure S4: Flow cytometric analysis for the transfection efficiency of Lipofectamine 2000/miR-34a and LA-OEI/miR-34a nanoparticles (*w/w*: 0.5) using Cy5-labeled miR-34a (A) and the quantitative measurement based on mean fluorescence intensity (MFI) (B). Data were presented as the mean value ± SD of triplicate experiments, Figure S5: Flow cytometric analysis for the endocytosis ability of FITC-labeled OEI and LA-OEI (A) and the quantitative measurement based on mean fluorescence intensity (MFI) (B). Data were presented as the mean value ± SD of triplicate experiments, Figure S6: Live/dead staining assay of HeLa cells after the treatment with different nanoparticles. The scale bar is 100 μm, Figure S7: The anti-proliferative effect after the miR-34a transfection. (A): The colony formation assay of HeLa cells after the treatment with different nanoparticles: (a) control, (b) PEI/miR-34a, (c) LA-OEI/miR-34a, (d) OEI/miR-34a, (e) miR-34a and (f) LA-OEI/N.C. The scale bar is 200 μm. (B): Quantitative analysis of the colony formation in HeLa cells after the treatment with different nanoparticles. Data were presented as the mean value ± SD of triplicate experiments, Figure S8: Flow cytometric analysis for the intracellular ROS level after the transfection of different nanoparticles for 48 h, using DCFH-DA as a probe (A) and the quantitative measurement based on mean fluorescence intensity (MFI) (B). Data were presented as the mean value ± SD of triplicate experiments, Figure S9: The relative activities of caspase-3, -8 and -9 in HeLa cells after the treatment with different nanoparticles. Data were presented as the mean value ± SD of triplicate experiments, Figure S10: Quantitative analysis for the G1 phase arrest of HeLa cells after miR-34a transfection. Data were presented as the mean value ± SD of triplicate experiments (* *p* < 0.05), Table S1: The hydrodynamic diameter and zeta potential values of LA-OEI/miR-34a and OEI/miR-34a nanoparticles.

## Sample Availability

Sample of the carrier LA-OEI is available from the authors.
