# Supplementary material for: Lipoic Acid-Modified Oligoethyleneimine-Mediated miR-34a Delivery to Achieve the Anti-Tumor Efficacy
Source: Molecules. 2021 Aug 10;26(16):4827. doi: 10.3390/molecules26164827 (PMC8400101; doi:10.3390/molecules26164827)
Supplement: Supplementary file 1 [file molecules-26-04827-s001.zip › molecules-1317649-supplementary.pdf]

Supporting Information

Lipoic acid-modified oligoethyleneimine-mediated miR-34a delivery to achieve the anti-tumor efficacy

Yu Huang, Longxiang Wang, Yingxuan Chen, Haobo Han,* Quanshun Li*

Key Laboratory for Molecular Enzymology and Engineering of Ministry of Education, School of Life Sciences, Jilin University, Changchun 130012, China

*Corresponding author.

Tel. and Fax: +86-431-85155200.

E-mail: quanshun@jlu.edu.cn (Q. Li); hanhaobo@jlu.edu.cn (H. Han).

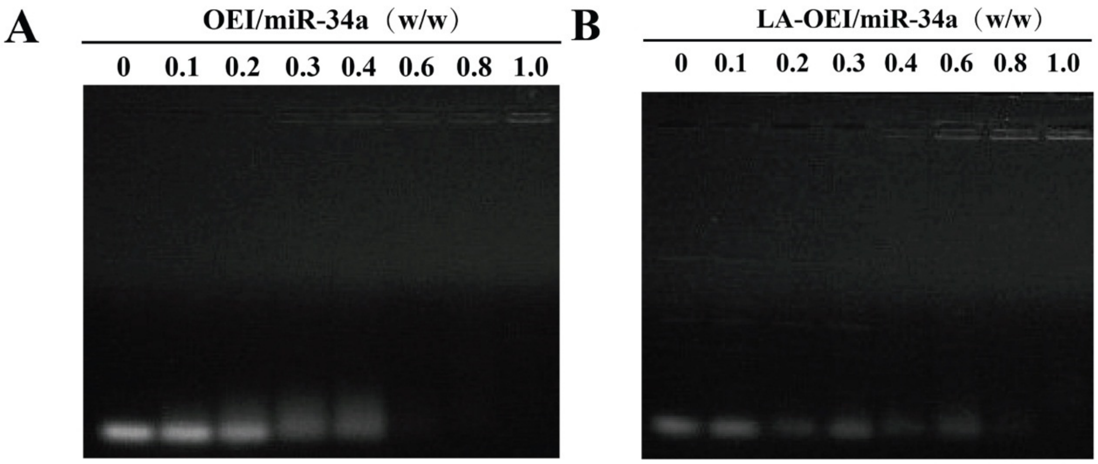

Figure S1. The miR-34a binding and condensation ability of different carriers using the gel retardation assay. A: OEI/miR-34a nanoparticles; B: LA-OEI/miR-34a nanoparticles.

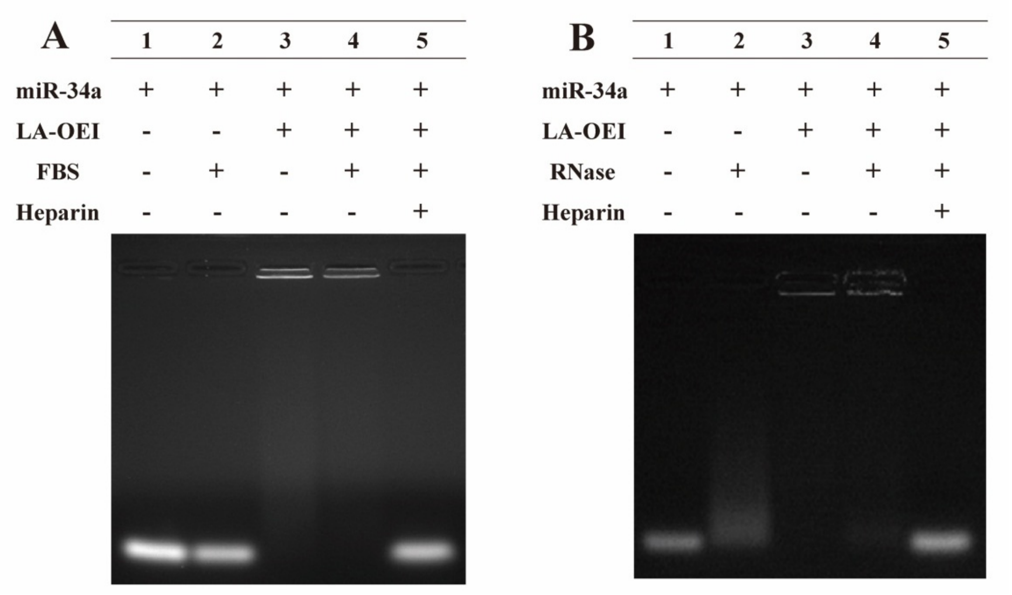

Figure S2. The protective effect of LA-OEI from the degradation of miR-34a. A: 50% FBS; B: RNase A.

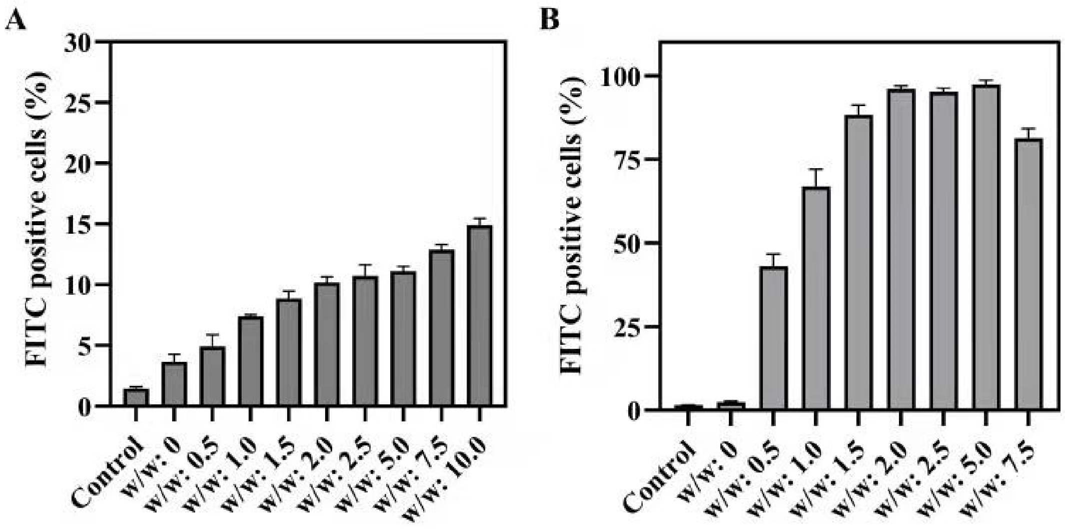

Figure S3. Quantitative analysis for the transfection efficiency of nanoparticles at different mass ratios based on FITC positive cells. A: OEI/miR-34a; B: LA-OEI/miR-34a. Data were presented as the mean value ± SD of triplicate experiments.

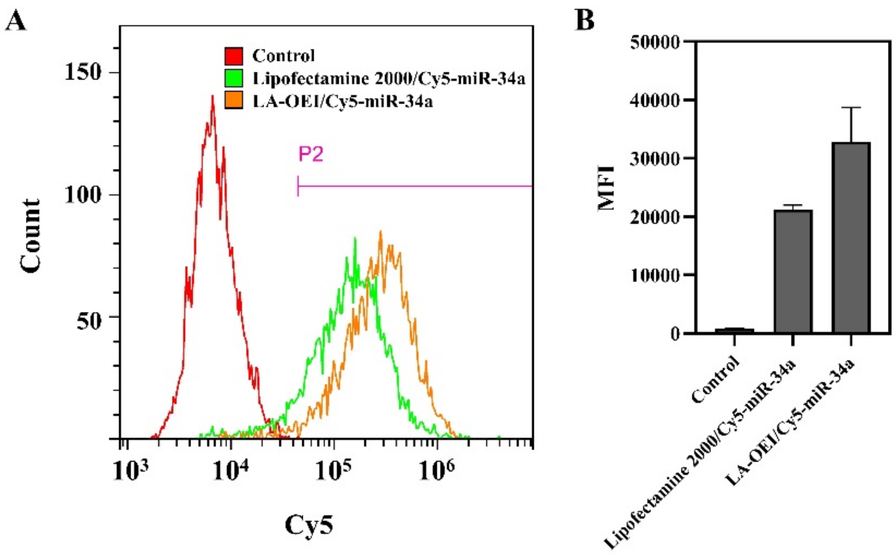

Figure S4. Flow cytometric analysis for the transfection efficiency of Lipofectamine 2000/miR-34a and LA-OEI/miR-34a nanoparticles (w/w: 0.5) using Cy5-labeled miR-34a (A) and the quantitative measurement based on mean fluorescence intensity (MFI) (B). Data were presented as the mean value ± SD of triplicate experiments.

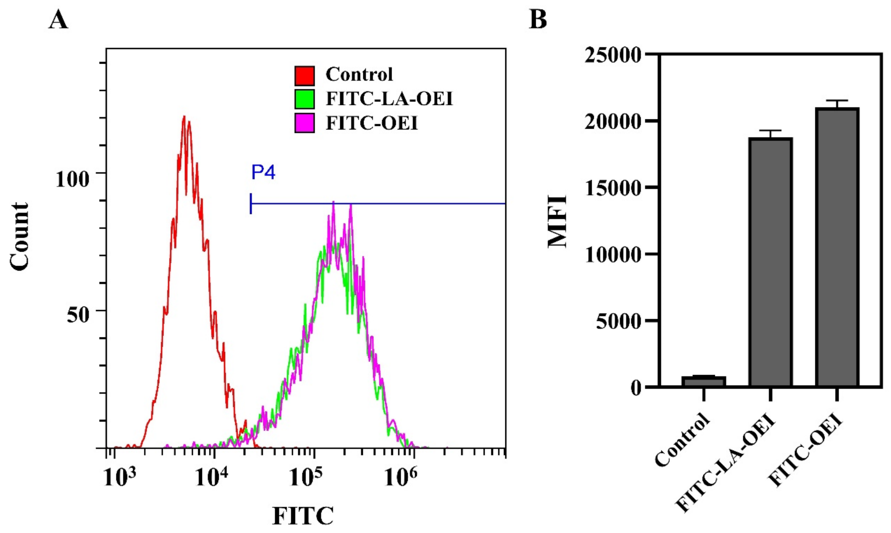

Figure S5. Flow cytometric analysis for the endocytosis ability of FITC-labeled OEI and LA-OEI (A) and the quantitative measurement based on mean fluorescence intensity (MFI) (B). Data were presented as the mean value ± SD of triplicate experiments.

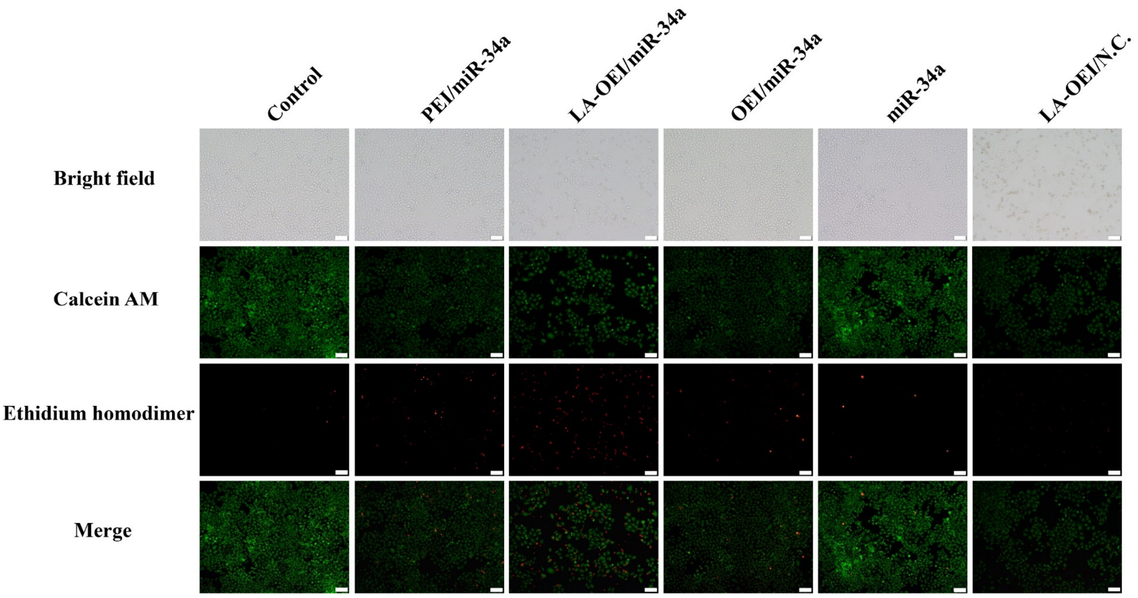

Figure S6. Live/dead staining assay of HeLa cells after the treatment with different nanoparticles. The scale bar is 100 μm.

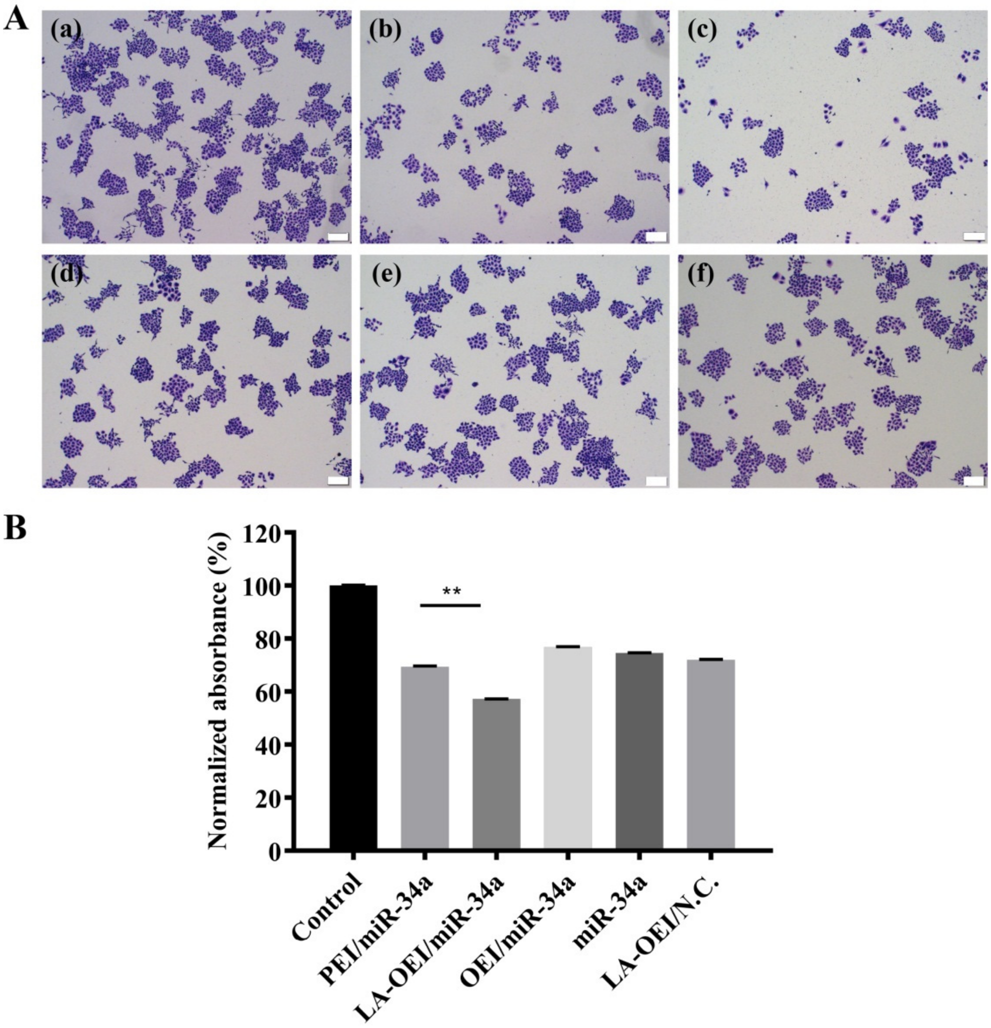

Figure S7. The anti-proliferative effect after the miR-34a transfection. A: The colony formation assay of HeLa cells after the treatment with different nanoparticles: (a) control, (b) PEI/miR-34a, (c) LA-OEI/miR-34a, (d) OEI/miR-34a, (e) miR-34a and (f) LA-OEI/N.C. The scale bar is 200 μm. B: Quantitative analysis of the colony formation in HeLa cells after the treatment with different nanoparticles. Data were presented as the mean value ± SD of triplicate experiments.

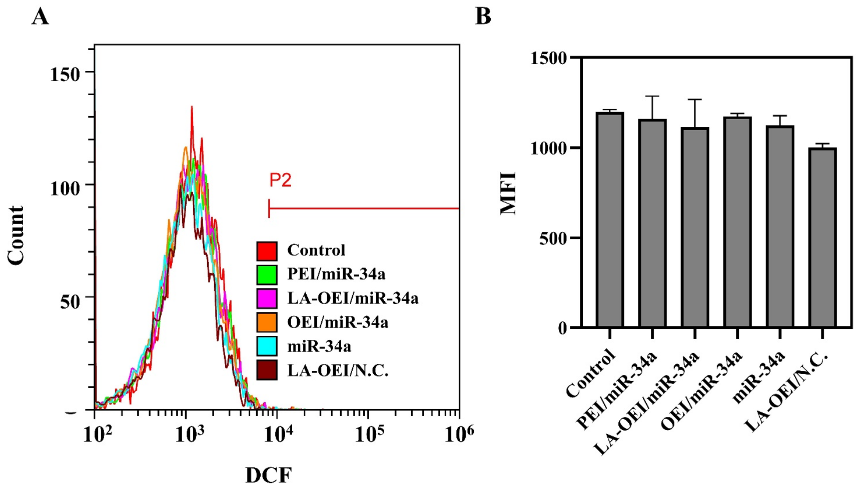

Figure S8. Flow cytometric analysis for the intracellular ROS level after the transfection of different nanoparticles for 48 h, using DCFH-DA as a probe (A) and the quantitative measurement based on mean fluorescence intensity (MFI) (B). Data were presented as the mean value ± SD of triplicate experiments.

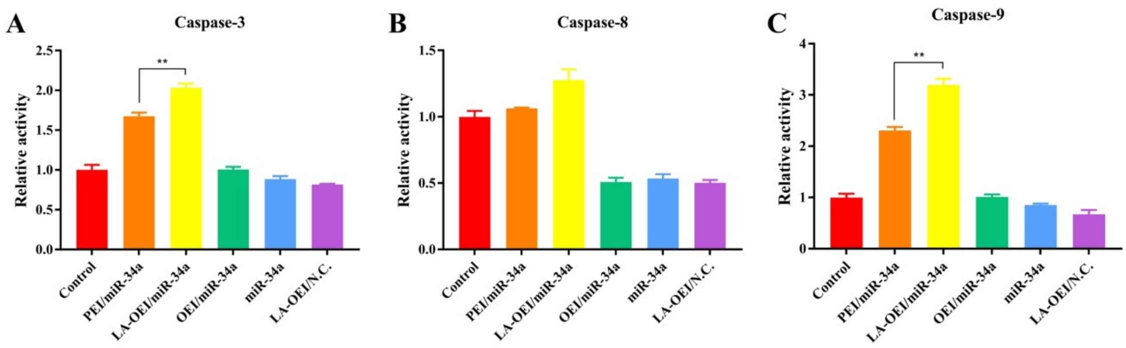

Figure S9. The relative activities of caspase-3, -8 and -9 in HeLa cells after the treatment with different nanoparticles. Data were presented as the mean value ± SD of triplicate experiments.

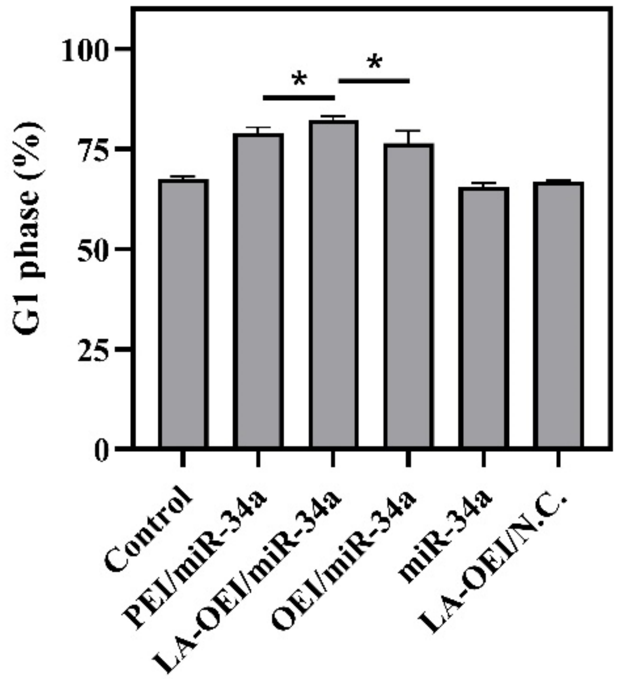

Figure S10. Quantitative analysis for the G1 phase arrest of HeLa cells after miR-34a transfection. Data were presented as the mean value ± SD of triplicate experiments (*p < 0.05).

Table S1. The hydrodynamic diameter and zeta potential values of LA-OEI/miR-34a and OEI/miR-34a nanoparticles.

Nanoparticles	Mass ratio	Hydrodynamic diameter (nm)	Polydispersity index (PDI)	Zeta potential (mV)
LA-OEI/miR-34a	0.5	293.2 ± 2.6	0.318	-12.0 ± 0.8
	1.0	288.9 ± 3.6	0.373	+10.5 ± 1.2
	2.0	197.7 ± 4.4	0.362	+17.1 ± 0.9
	3.0	187.8 ± 1.2	0.316	+25.8 ± 0.6
	4.0	138.7 ± 0.9	0.234	+30.2 ± 0.9
	5.0	123.4± 2.1	0.276	+47.0 ± 0.7
OEI/miR-34a	0.5	386.9 ± 8.8	0.386	-15.0± 0.3
	1.0	280.0 ± 7.8	0.244	-0.1 ± 0.8
	2.0	218.5 ± 6.3	0.113	+5.3 ± 0.4
	3.0	190.2± 2.3	0.199	+8.2 ± 0.7
	4.0	181.9 ± 1.9	0.127	+13.6 ± 0.5
	5.0	167.0 ±2.0	0.131	+17.5 ± 0.7
